# A PEGDA/DNA Hybrid Hydrogel for Cell-Free Protein Synthesis

**DOI:** 10.3389/fchem.2020.00028

**Published:** 2020-02-18

**Authors:** Jinhui Cui, Dan Wu, Qian Sun, Xiuzhu Yang, Dandan Wang, Miao Zhuang, Yiheng Zhang, Mingzhe Gan, Dan Luo

**Affiliations:** ^1^CAS Key Laboratory of Nano-Bio Interface, Suzhou Institute of Nano-Tech and Nano-Bionics, Chinese Academy of Sciences, Suzhou, China; ^2^School of Nano-Tech and Nano-Bionics, University of Science and Technology of China, Hefei, China; ^3^School of Pharmacy, Xi'an Jiaotong University, Xi'an, China; ^4^PLD Technology Co., Ltd., Suzhou, China; ^5^Central Laboratory, School of Medicine, Renji Hospital, Shanghai Jiao Tong University, Shanghai, China; ^6^State Key Laboratory of Oncogenes and Related Genes, Shanghai Cancer Institute, School of Medicine, Renji Hospital, Shanghai Jiao Tong University, Shanghai, China; ^7^Department of Biological and Environmental Engineering, Cornell University, Ithaca, NY, United States; ^8^Kavli Institute at Cornell for Nanoscale Science, Cornell University, Ithaca, NY, United States

**Keywords:** hybrid hydrogel, cell-free protein synthesis, chemical cross-linking, DNA hydrogel, PEGDA

## Abstract

Cell-free protein synthesis (CFPS) has the advantage of rapid expression of proteins and has been widely implemented in synthetic biology and protein engineering. However, the critical problem limiting CFPS industrial application is its relatively high cost, which partly attributes to the overexpense of single-use DNA templates. Hydrogels provide a possible solution because they can preserve and reutilize the DNA templates in CFPS and have great potential in elevating the protein production yield of the CFPS. Here, we presented a low-cost hybrid hydrogel simply prepared with polyethylene glycol diacrylate (PEGDA) and DNA, which is capable of high-efficient and repeated protein synthesis in CFPS. Parameters governing protein production specific to hybrid hydrogels were optimized. Structures and physical properties of the hybrid hydrogel were characterized. Transcription and expression kinetics of solution phase system and gel phased systems were investigated. The results showed that PEGDA/DNA hydrogel can enhance the protein expression of the CFPS system and enable a repeated protein production for tens of times. This PEGDA/DNA hybrid hydrogel can serve as a recyclable gene carrier for either batch or continuous protein expression, and paves a path toward more powerful, scalable protein production and cell-free synthetic biology.

## Introduction

Cell-free protein synthesis (CFPS) is an *in vitro* life simulation system that synthesizes proteins using cell extracted machinery, exogenous substrates, and DNA or RNA templates without the constraint of cells. CFPS is a versatile technology and has been widely applied in the field of biological research. CFPS systems can use linear DNA template amplified by PCR for protein expression and realize rapid and parallel expression of multiple target genes. The open nature of the CFPS system allows unique environmental control and freedom of design, thus enabling simple and efficient protein production, as well as synthesis of proteins that are difficult to express in living cells, such as transmembrane proteins and toxic proteins (Lim et al., 2016; Henrich et al., 2017; Thoring et al., 2017). It is likely that in the near future, the toolkit of CFPS systems can be further expanded and optimized to facilitate the expression of any desired proteins. In addition, cell-free reactions are scalable, ranging from microliter to liter scale (Zawada et al., 2011). Scaling up the cell-free protein production system to the liter scale is the basis for large-scale production of pharmaceutical relevant proteins (Stech et al., 2013).

Although CFPS technology has progressed rapidly over the past decade, there are still some challenges to overcome. The problem of high cost and low production yield limited its application in industry. In order to improve the life time of the template and the yield of protein for CFPS, many efforts have been taken from the perspective of inhibiting the activity of the nuclease, preparing an extract from genetically modified strains, improving energy regeneration and byproducts recycling, etc. (Caschera and Noireaux, 2014; Fujiwara and Doi, 2016; Schoborg et al., 2016). These efforts, indeed, improved the protein production of CFPS to a certain degree. But vast DNA consumption in large-scale reactions is still one of the bottlenecks in limiting the development of CFPS industrialization. Hydrogels-incorporated gene templates provided a possible solution to this problem.

Hydrogels are three-dimensional polymeric networks made of highly hydrophilic monomers (Hoare and Kohane, 2008; Pan et al., 2013; Wei et al., 2016; Glass et al., 2018). In the past few decades, numerous hydrogels have been developed based on natural and synthetic molecules such as cellulose, chitosan, polypeptide and poly(acrylic acid), poly(ethylene glycol), and poly(ethylene glycol) diacrylate (PEGDA).

DNA is a natural polymer material that possesses many unique and fascinating properties, including intrinsic genetic functions, broad biocompatibility, precise molecular recognition capability, tunable multifunctionality, and convenient programmability. DNA can be used as the only component of a hydrogel or a cross-linker connecting the main building blocks to form hybrid hydrogels through chemical reactions or physical entanglement. The application of DNA hydrogels has drawn much attention in recent years. For instance, target stimuli-responsive DNA hydrogels were engineered based on DNA aptamers that cross-linked with linear polyacrylamide chains to sense changes of pH, temperature, or the concentration of metabolite and release their load as a result of such a change (Yang et al., 2008). DNA hydrogels were developed as a platform for controlled release delivery of antigens due to their highly efficient cross-linking maintenance in a physiological environment, which allows *in situ* encapsulation and preservation of payloads (Nishikawa et al., 2014).

We previously invented a DNA hydrogel that was termed as P-gel for CFPS. P-gel exhibited great potential in elevating protein production efficiency, yield, and reusability (Park et al., 2009a,b; Kahn et al., 2016). To further reduce the DNA consumption in protein producing hydrogel, in this study, we successfully constructed a PEGDA/DNA hybrid hydrogel for CFPS. The cost of PEGDA/DNA hybrid hydrogel was reduced more than 30 times compared to that of P-gel. The optimized PEGDA/DNA hybrid hydrogel enhanced protein production 22.7-fold over the solution phased reactions. The characterization, transcription, and expression kinetic studies provided an insight into the mechanism of the protein production enhancement. Moreover, it was proved that the PEGDA/DNA hybrid hydrogel can be reused 10 times in CFPS, which showed great potential in large-scale CFPS application.

## Materials and Methods

### Materials

All chemicals, unless otherwise stated, were purchased from Sigma-Aldrich and were used as received. Bacteria *Escherichia coli* BL21 (DE3) strain was preserved in our laboratory. Plasmid pID-sfGFP was reconstructed from a gift plasmid pIJ8660 from Professor Lixin Zhang (East China University of Science and Technology) with a fragment deletion from restriction sites *Nhe*I to *Age*I. Plasmid pUTI-beacon was constructed by replacing the sfGFP gene of pID-sfGFP with the gene coding a urinary trypsin inhibitor protein Bikunin. All primers including 5′ acrydite modified primers and molecular beacon oligos were synthesized by Synbio Technologies. High-fidelity Pfu DNA polymerase for PCR amplification was purchased from Beyotime Biotechnology.

### Preparation of PEGDA/DNA Hybrid Hydrogel

The DNA part for the construction of PEGDA/DNA hybrid hydrogel was prepared by PCR with 5′ acrydite modified primers F1/R1 (Table 1). Plasmid pID-sfGFP was used as the template for PCR amplification. The reactions contained 1 × high-fidelity PCR master mix with 1 × Pfu buffer (Beyotime). Thermocycling for PCR was 30 s at 98°C for the initial denaturation followed by 10 cycles of 10 s at 95°C, 30 s at 65°C, and 30 s at 72°C and a final extension of 5 min at 72°C. The amplified DNA product was purified with Cycle Pure kit (Omega, Bio-tek) and stored at −20°C before use. The DNA template in the hybrid hydrogel for real-time measurement of mRNA transcription was prepared in the same way as 5′ acrydite modified primers F2/R2 (Table 1) and plasmid pUTI-beacon as the PCR template.

**Table 1:** Primer and molecular beacon sequences.

**Primer**	**Sequence**
F1	5′-Acrydite-TGGAGCGGATCGGGGATTGT-3′
R1	5′-Acrydite-CCGGTCGACTCTAGCTAGAG-3′
F2	5′-Acrydite-ACGTAACTCTAACGTTGACCGGCTGCAGCCC-3′
R2	5′-Acrydite-AGAGTTACGTTGAGAGAGTTAAGCTTGAATTCGG ATCCTTACAGC-3′
Beacon target	5′-AACTCTCTCAACGTAACTCTCTCAACGT-3′
Beacon probe	5′-FAM/mCmCmGmCmAmAmAmAmCmGmUmUmGmAmGm AmGmAmGmAmUmAmAmGmCmGmG-BHQ1-3′

To prepare the PEGDA/DNA hybrid hydrogel, PEGDA (M_n_ = 575) was mixed with acrydite modified linear DNA PCR product at predetermined percentage and concentration, respectively. After fully mixing, a certain amount of ammonium persulfate (APS) and tetramethylethylenediamine (TEMED) were added at equimolar concentrations to start the reaction and were polymerized at room temperature for 3 h unless specified to form the PEGDA/DNA hybrid hydrogel. After the reaction, the hydrogel was soaked and washed with 1 × PBS buffer and absolute ethanol three times, respectively, to remove the partially reacted monomers and excessive initiators in the hydrogel.

### Scanning Electron Microscopy

For scanning electron microscopy (SEM), the hydrogel was freeze-dried for 24 h and brittle-fractured with liquid nitrogen to obtain its cross section. After Au-sputter coating, the microstructure of the freeze-dried hydrogel was observed with field emission scanning electron microscope (FE-SEM S4800, Hitachi).

### Swelling Tests of PEGDA/DNA Hybrid Hydrogel

Swelling tests were conducted by using a gravimetric method. Lyophilized hydrogels with confirmed weights were immersed in the CFPS reaction buffer solution (described below). At selected time intervals, the hydrated gels were taken out and wiped with a filter paper to remove excess water from the gel surface and were then weighed. The swelling ratio (SR) was calculated according to the following equation:

$$\begin{array}{l} \text{SR}=\;W_{\text{t}}/W_{\text{d}} \end{array}$$

where *W*_d_ and *W*_t_ denote the weight of the dried hydrogel and the weight of the swollen hydrogel, respectively.

### Rheological Characterization of Hydrogel

For rheological measurements, the hydrogel was prepared at 100-μl scale with and without the component of DNA (100 ng/μl). The pre-gel mixture was added to a round shape mold matched with the parallel plate of the rheometer as prepared. After gelation, the gel was taken out from the mold and rheological characterization was conducted with a rheometer (Kinexus pro+). An 8-mm parallel-plate geometry and a gap of 2 mm are used for all experiments, which are done at 25°C. A solvent trap is used to prevent water evaporation during the measurements. Frequency sweep measurements are carried out over the frequency range 0.1–10 Hz, and a deformation amplitude γ0 = 0.01 is selected to ensure that the oscillatory deformation is within the linear regime.

### Cell-Free Protein Production With DNA Hydrogels

The crude extract was prepared as described in Caschera and Noireaux (2014) with slight modifications. *E. coli* BL21 Rosetta2 DE3 strain was used as the lysate source. Besides, isopropyl-thiogalactopyranoside (IPTG, 1 mM) was added to the culture media to induce the production of T7 RNA polymerase when culture OD600 reached 0.6. The cell-free reaction buffer was composed of 50 mM HEPES (pH 8), 1.5 mM ATP and GTP, 0.9 mM CTP and UTP, 0.2 mg/ml tRNA, 0.26 mM coenzyme A, 0.33 mM NAD, 0.75 mM cAMP, 0.068 mM folinic acid, 1 mM spermidine, 30 mM 3-PGA, 1 mM DTT, 2% PEG8000, and 3 mM of each of the 20 amino acids. The Mg-glutamate and K-glutamate concentrations were 4 and 80 nM, respectively.

The cross-linked PEGDA/DNA hybrid hydrogel containing a certain amount of PEGDA and linear DNA was used for cell-free protein production in a solution containing 6.67 μl of cell-free lysate, 10.73 μl of the reaction buffer, and nuclease-free water. Two microliters of the hydrogel was used for CFPS. The reaction volume was 20 μl unless stated otherwise. The reactions were incubated at 30°C in a thermomixer (Eppendorf Thermomixer C) with 1,000 rpm for 15 h. End-point fluorescence was measured with a plate reader (BioTek synergy/H1) and was used to determine the concentration of GFP products according to a GFP standard curve (Figure S3). For the reuse of hybrid hydrogel, after each cycle of reaction, the reaction tube was centrifuged at 1,500 rpm for 3 min, and the supernatant was pipetted out and stored for quantification. The remainder of the hydrogel was washed three times with 1 × PBS and added to a fresh lysate solution for CFPS reactions.

### Real-Time Measurement of mRNA Transcription

The real-time transcription of mRNA in CFPS reaction was measured with a molecular beacon. The beacon probe bonds to a two-time repeat target sequence (Table 1) tagged after the UTI gene once the mRNA was transcribed. One hundred micromolar beacon probe was annealed with a procedure of incubation under a gradient temperature from 95°C to 25°C and stored at 4°C before use. The beacon probe was added to the CFPS reaction at a final concentration of 1 μM, and the reactions were conducted in 96-well plates at 30°C and monitored in real time for 6 h by a plate reader (BioTek synergy/H1).

## Results and Discussion

### Principle of PEGDA/DNA Hybrid Hydrogel

**Figure 1:**
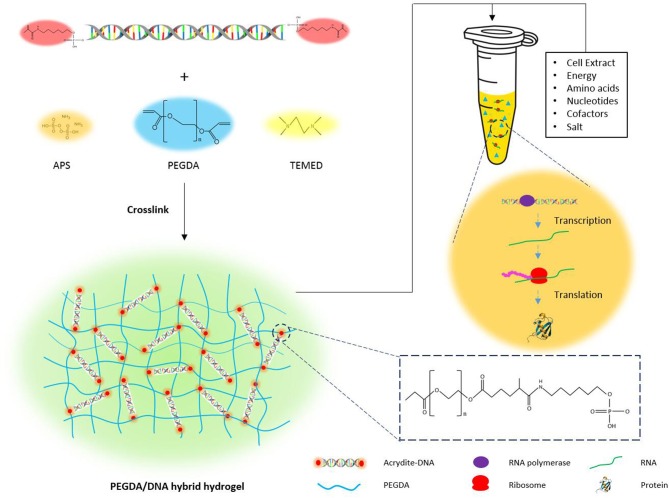
The schematic illustration of the preparation of PEGDA/DNA hydrogel and its use for cell-free protein production.

The principle for the design of the PEGDA/DNA hybrid hydrogel was to prepare a hydrogel that not only can efficiently express the protein of interests in a cell-free system but also can be easily recovered from the reaction solution in a batch reaction mode or can serve as a long-term template in a continuous reaction mode. A schematic illustration of the preparation process and the structure model of the PEGDA/DNA hybrid hydrogel is shown in Figure 1. First, to prepare the hybrid hydrogel DNA component, which contained the gene template for protein expression, a pair of 5′-acrydite modified oligonucleotide primers was used to PCR amplify linear DNA from a plasmid (Figure S1). The amplified DNA contains acrydite groups on its both ends and includes all the necessary transcription elements for protein expression *in vivo*, such as a promoter, a ribosome binding site, the gene of interest (*sfgfp* for latter optimization), and a translation terminator. Then, the acrydite modified DNA was mixed with PEGDA. The acrydite group on DNA and the acrylate group on PEGDA were cross-linked and polymerized under the free radical induced addition reaction, when the radical initiator ammonium persulfate (APS) and catalyst tetramethylethylenediamine (TEMED) were added. The resultant hydrogel can be regarded as a PEGDA/DNA hybrid network structure where linear DNA immobilized on the gel matrix. The hydrogel was then submerged in a CFPS system containing cell extract, energy, substrates, salts, and other cofactors needed for protein synthesis to facilitate protein expression in a cell-free manner.

### Optimization of PEGDA/DNA Hybrid Hydrogel for CFPS

As a free radical induced reaction product, the hybrid gel mechanical property and functionality were correlated with the reaction parameters. Different reaction parameters resulted diverse mechanical properties and performances of hybrid hydrogels in CFPS. We investigated and optimized the parameters governing protein production that were specific to PEGDA/DNA hybrid hydrogels. These parameters included the concentration of PEGDA, the concentration of APS, reaction gelation time and the amount of DNA in the gel scaffolding.

**Figure 2:**
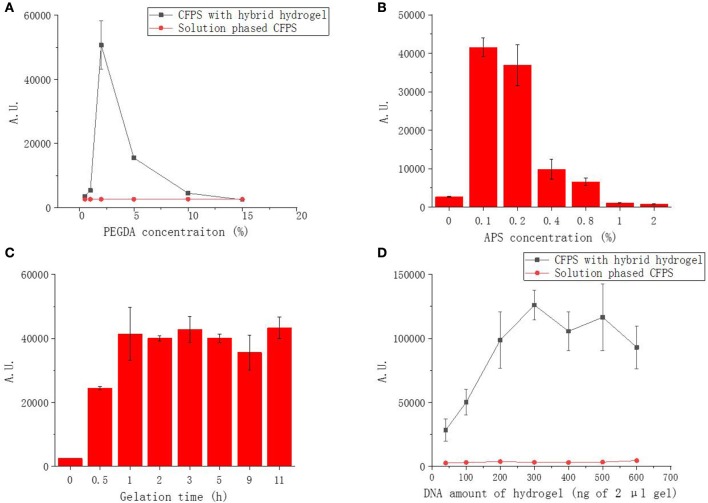
Functional sfGFP expression from PEGDA/DNA hybrid hydrogel phased CFPS with different parameters. **(A)** Effect of PEGDA concentration on fluorescent protein expression. **(B)** Effect of APS concentration on fluorescent protein expression. **(C)** Effect of gelation time on fluorescent protein expression. **(D)** Effect of DNA amount in gel on fluorescent protein expression.

We first optimized the PEGDA concentration of the hybrid hydrogel with fixed DNA amount at 100 ng for 2 μL hybrid hydrogel in each CFPS reaction. The protein expression results demonstrated that, with the PEGDA concentration ranging from 0.5 to 15%, PEGDA/DNA hybrid hydrogels exhibited overall higher efficiency and better yield under most of PEGDA concentrations compared to the solution phased reactions with linear DNA template (Figure 2A). However, the protein yield increased dramatically when the PEGDA concentration increased from 0.5 to 2% and decreased significantly when the PEGDA concentration decreased from 2 to 5%. Two percentage of PEGDA in hybrid hydrogel exhibited most efficient protein production and the protein yield was 270 μg/mL and represented 9.5-fold enhancement over the solution phased reaction. The increase of protein expression at lower PEGDA concentration can be explained by the improved gene stability, high local concentration and fast turnover rate of enzymes in gel format CFPS reactions (Park et al., 2009b; Yang et al., 2013; Guo et al., 2019). While the decrease of protein expression with higher PEGDA concentration could be possibly due to the small pore size of the gel caused by high density of PEGDA. Too small pore size hindered enzymes interacted with the gene inside the gel, thus stopped the gene transcription.

To investigate the effect of APS concentration to the performance of the hybrid hydrogel in CFPS, we fixed the PEGDA and DNA concentration of the gel (2% and 100 ng in 2 μL gel) and varied the APS concentration used in the preparation of hybrid hydrogel from 0 to 2%. As shown in Figure 2B, the expression of sfGFP was highest at 0.1% and decreased dramatically when the APS concentration more than 0.2%. APS functioned as an initiator to generate free radicals which leads to the crosslinking reaction in the gelling system. The higher the concentration of APS, the shorter time required for the gelation and the higher density of cross-linking. At lower APS concentration, the performance of the gel was improved because of the same reason of improved gene stability, high local concentration and fast enzyme turnover rate as at low PEGDA concentration. The decrease of protein expression with higher APS concentration was possibly due to the damage of DNA during gel formation as higher APS concentration provided more free radical which was a common factor causing damage to DNA molecules, resulting that the template could not be transcribed and translated, and the fluorescent protein could not be further expressed.

We noticed that gelation time could affect the gel formation and eventually affect hydrogel functionality in CFPS. Thus, we further optimized the gelation time effect to the protein production of CFPS with hybrid hydrogels formed under different gelling times. At fixed PEGDA, APS and DNA concentration (2%, 0.1%, and 100 ng in 2 μL gel), indeed, the protein production yield increased with prolonged gelation time at the first hour and became stable when the gelation time was more than 2 h (Figure 2C).

The above results demonstrated that the PEGDA/DNA hybrid hydrogel could efficiently enhance the protein production in CFPS. To further optimize the protein production yield with PEGDA/DNA hybrid hydrogel, we explored the gene amount effect by varying the DNA amount in the hydrogel while keeping the concentration of PEGDA, APS and gelation time constant (2%, 0.1%, and 3 h). Compared to the solution phased CFPS reaction, the hydrogel system constantly enhanced protein expression in all tested DNA amounts and the protein expression reached plateau with 300 ng of DNA in 2 μL gel (equal to a concentration of 15 ng/μL of solution phased CFPS reaction) (Figure 2D). In particular, 300 ng DNA produced 691 ng/μL sfGFP protein representing 22.7-fold enhancement over the solution phased reaction. The protein production yield was slightly decreased when the DNA amount more than 500 ng. This could be attribute to the crowding effect which too crowd molecule environment hindered the diffusion and shuttling of enzymes in gel format of CFPS reaction (Guo et al., 2019).

### Characterization of Functional PEGDA/DNA Hybrid Hydrogel

From the above results, we have seen that PEGDA/DNA hybrid hydrogel can produce protein with higher efficiency and yield compared with solution phased CFPS. To understand the mechanism of the improvements, we decided to further study the characteristics of functional hybrid hydrogels with the PEGDA concentrations of 2% and 5% as they showed significant protein production improvements in CFPS reactions.

**Figure 3:**
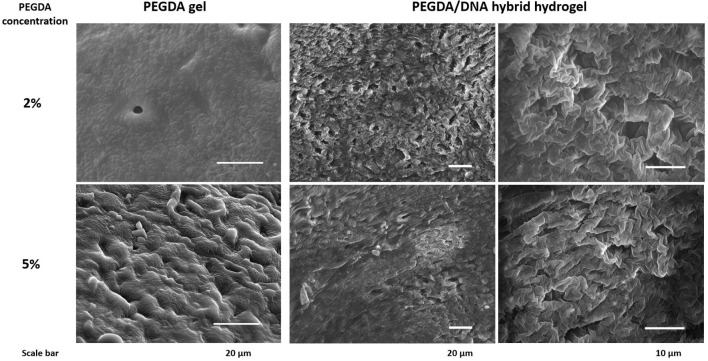
Scanning microscopy images of hydrogel with/without DNA and different concentration of PEGDA.

**Figure 4:**
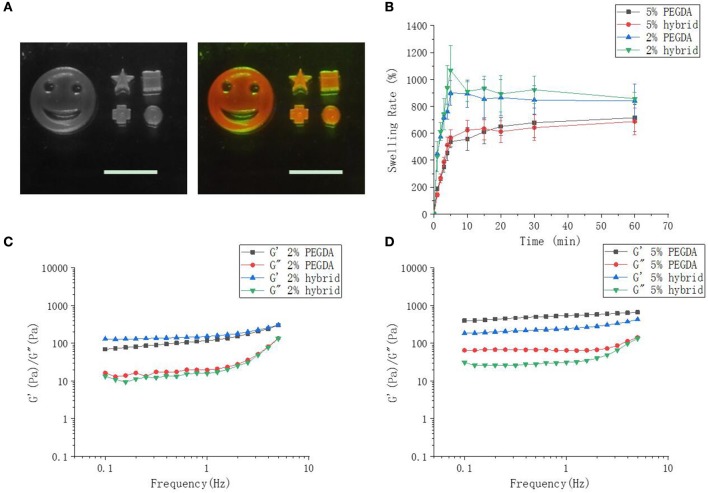
Hybrid hydrogel, swelling and rheology characterizations. **(A)** Images of different patterned hybrid hydrogel with 2% of PEGDA. The scale bar is 1 cm. The right image shows fluorescent gel stained with GelRed. **(B)** Swelling rate of 2 and 5% of hybrid hydrogel in CFPS reaction buffer. **(C,D)** Rheological characterization of 2% and 5% hydrogels.

Scanning microscopy images were used to reflect the three-dimensional network structure of hydrogels with/without DNA and different PEGDA concentrations. Figure 3 showed that with the incorporation of DNA to their gel networks, both 2 and 5% hydrogel become more porous than the gel with PEGDA only. In addition, hybrid hydrogels with 2% of PEGDA were more uniform than that with 5%, which indicated lower PEGDA concentration led to a more homogeneous network. Besides that, the pore size of 2% hydrogel was bigger than that of 5%. These results could partially explain the reason that the hybrid hydrogel with 2% of PEGDA showed a better performance in protein production than 5%. Hydrogel with 5% of PEGDA had a denser gel structure which might hinder the diffusion and shuttling of enzymes, substrates and reaction intermediates inside of the gel when doing CFPS and thus lowered the protein production efficiency. The photographs of patterned PEGDA/DNA hybrid hydrogel with 2% of PEGDA were shown in Figure 4A. The gel was stained with DNA-specific dye GelRed and the fluorescent image indicated the incorporation of DNA within the hydrogel.

The swelling of a cross-linked polymer by solvent is often used to assess cross-linking density (Oh et al., 1998). Swelling tests demonstrated that 5% hydrogel had a greater cross-linking density. With the extension of immersion time in CFPS reaction buffer, the swelling rate of two hybrid hydrogel increased sharply and 2% hydrogel swelled approximate 800%, while 5% hydrogels swelled approximate 600%. The biggest SR of 5% hydrogel was smaller than that of 2% (Figure 4B). The lower PEGDA content, the smaller the cross-linking density, and the greater swelling rate of the hydrogel. The addition of DNA to the gel networks did not change the swelling properties of the hydrogels.

Cross-linking density determines the mechanical property of the hydrogel (Hong et al., 2015). Low cross-linking density often results a soft gel with more elasticity, while high cross-linking density leads to a tough gel with less elasticity. By changing the concentration of PEGDA, we obtained a series of hybrid hydrogels with different stiffness (data not shown). However, hydrogels for CFPS should neither too soft which could be smashed by the shearing force during CFPS reaction nor too tough and dense which have already been proved by above results of lower protein production efficiency. In order to better understand the physical characteristics of the hybrid hydrogels, we further investigated the rheological properties of the hydrogels as they can reflect molecular motions in polymer sensitively (Li et al., 2017). The storage moduli G' and loss moduli G” of 2 and 5% hydrogels as a function of angular frequency are detected by frequency sweep measurements on a rheometer. The results showed that in either 2 or 5% hydrogels with or without DNA, the storage moduli G' of all hydrogels were obviously dependent on frequency and slightly increased with the augment of frequency (Figures 4C,D). The storage modulus in the low frequency region responding to frequency reflected an entanglement gel network or other physical networks (Rubinstein and Colby, 2003). On the other hand, the loss modulus G” of the hydrogels were also dependent on frequency. Loss modulus represents the energy dissipation capacity during deformation and the dependence of G” on frequency indicated that our hydrogels were viscoelastic rather than elastic. Moreover, the dependence degree of G' was close to 0 and G' was higher than G” indicated that chemical cross-linking still dominated though physical entanglement constituted part of the gel network. The addition of DNA to 2% hydrogel did not change the rheological properties of the hydrogel too much compared to that to 5%.

### Transcription and Expression Kinetics of Hybrid Hydrogel Phased CFPS

The characterization study of functional hybrid hydrogels showed evidences of their intrinsic properties were conducive to facilitate protein expression. We next employed molecular beacon and fluorescent protein sfGFP to study the kinetics of hydrogel phased cell-free gene transcription and protein expression. The mRNA of a non-fluorescent protein Bikunin was transcribe from the hybrid hydrogel with a 4-time repeat molecular beacon target to monitor the transcription kinetics. Bikunin is a serine protease inhibitor found in the blood serum and urine of humans and other animals. It is also known as urinary trypsin inhibitor (UTI) which is a pharmaceutical protein for the treatment of acute pancreatitis and other diseases (Pugia and Lott, 2005). The main source of pharmaceutical Bikunin is human urine and the recombinant protein is hard to be expressed in living cells with traditional protein expression method. The mRNA yield of UTI were measured in real-time by plate reader with the addition of a molecular beacon probe, which was a hairpin-shaped oligonucleotide probe (Guo et al., 2019). The fluorophore and quencher modified beacon probe was linearized by hybridizing to the mRNA sequence of UTI and then led a fluorescent signal. Protein expression kinetics was conducted by measuring the fluorescent signal of sfGFP instead of molecular beacon in a similar approach.

**Figure 5:**
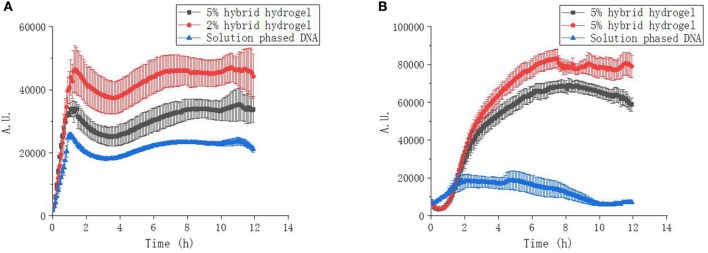
Transcription and expression kinetics of CFPS reactions. **(A)** UTI transcription kinetics of hybrid hydrogels and solution phased CFPS reactions. **(B)** sfGFP expression kinetics of hybrid hydrogels and solution phased CFPS reactions.

The transcription kinetic curves showed, at the same amount of gene template, the transcription of mRNA from hybrid hydrogels were always higher than that from solution phased linear DNA (Figure 5A). This indicated that the up-regulation of protein expression with PEGDA/DNA hybrid hydrogel was due to the enhancement of mRNA transcription and spatial localization of gene template can accelerate the transcription speed and increase the mRNA yield. Hydrogel with 2% PEGDA had an even higher mRNA transcription level than hydrogel with 5%. This was probably due to the high cross-linking density of 5% hydrogel caused steric hindrance for RNA polymerase and other transcription factors. Similar results were observed for sfGFP expression kinetics (Figure 5B). But to our surprise, solution phased reaction showed stronger expression at the initial stage of the experiment which indicated that the translation efficiency of solution phased reaction was higher than that of hydrogel phased reactions. This was reasonable because that translation machinery and substrates were homogenously distributed over the entire reaction environment. To accomplish the translation process, either the mRNA needed to diffuse out of the hydrogel or the translation machinery and substrates required to shuttle and diffused into the hydrogel in the gel phased reaction. More importantly, proteins expressed in hydrogel phased CFPS were totally soluble and functional (Figure S2).

### Reuse of PEGDA/DNA Hybrid Hydrogel for CFPS

**Figure 6:**
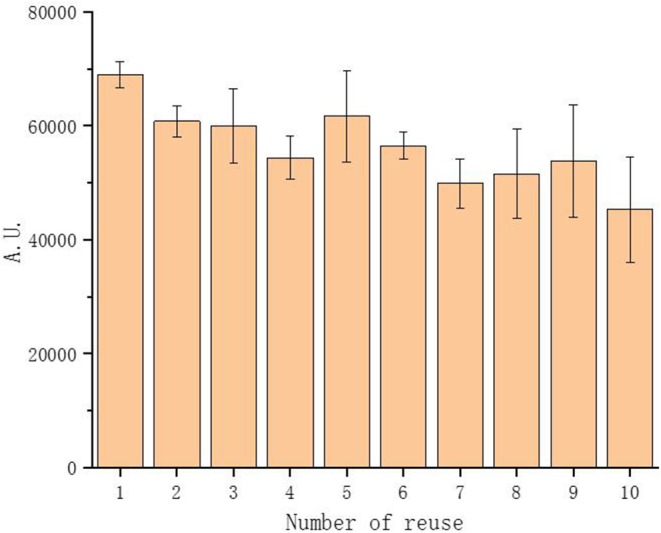
Reuse of PEGDA/DNA hybrid hydrogel.

Traditional CFPS utilize solution phased DNA as protein expression template. One of the problems using solution phased DNA for CFPS is that the template DNA are more likely to be lost due to degradation and adhesion on the reaction vessel wall, resulting in low protein production yield and large amounts of gene demand for numerous batches of reactions. On the other hand, hydrogels with the mount of gene templates inside the gel matrix appeared to be protected against degradation due to the characteristic structures and can be easily recovered by a simple centrifugation from lysates after expression for reuse. To confirm that, we tested our PEGDA/DNA hybrid hydrogel as a recyclable gene carrier for repeated protein production with CFPS. As expected, the hybrid hydrogel can successfully be reused up to 10 times without significant loss on protein production yields (Figure 6).

## Conclusion

In conclusion, we presented a strategy for constructing a hybrid hydrogel integrating cross-linking chemicals, PEGDA in our case, and DNA *via* free radical induced polymerization. The PEGDA/DNA hybrid hydrogel can enhance protein expression of CFPS system and serve as recyclable gene carrier for repeated protein production. We believed that, with further exploitation, this PEGDA/DNA hybrid hydrogel can be developed as a platform and applied to large scale cell-free protein production in either batch or continuous mode. The development of PEGDA/DNA hybrid hydrogel paved a path toward more powerful, scalable protein production and cell-free synthetic biology.

## Data Availability Statement

The datasets generated for this study are available on request to the corresponding author.

## Author Contributions

JC and DWu designed and conducted the experiments, performed data analysis, and prepared the manuscript. QS, XY, DWa, MZ, and YZ assisted in designing and performing experiments. MG and DL supervised the study. All authors contributed to the writing of the manuscript.

### Conflict of Interest

XY and MZ are employed by PLD Technology Co., Ltd. The remaining authors declare that the research was conducted in the absence of any commercial or financial relationships that could be construed as a potential conflict of interest.

## References

[B1] Caschera F. Noireaux V. J. B. (2014). Synthesis of 2.3 mg/ml of protein with an all *Escherichia coli* cell-free transcription–translation system. Biochimie 99, 162–168. 10.1016/j.biochi.2013.11.025 24326247

[B2] Fujiwara K. Doi N. (2016). Biochemical preparation of cell extract for cell-free protein synthesis without physical disruption. PLoS ONE 11:e0154614. 10.1371/journal.pone.0154614 27128597 PMC4851396

[B3] Glass S. Trinklein B. Abel B. Schulze A. (2018). TiO2 as photosensitizer and photoinitiator for synthesis of photoactive TiO_2_-PEGDA hydrogel without organic photoinitiator. Front. Chem. 6:340. 10.3389/fchem.2018.00340 30131954 PMC6090817

[B4] Guo X. C. Bai L. H. Li F. Huck W. T. S. Yang D. Y. (2019). Branched DNA architectures produced by PCR-based assembly as gene compartments for cell-free gene-expression reactions. ChemBioChem. 20, 2597–2603. 10.1002/cbic.201900094 30938476

[B5] Henrich E. Sormann J. Eberhardt P. Peetz O. Mezhyrova J. Morgner N.. (2017). From gene to function: cell-free electrophysiological and optical analysis of ion pumps in nanodiscs. Biophys. J. 113, 1331–1341. 10.1016/j.bpj.2017.03.026 28450130 PMC5607034

[B6] Hoare T. R. Kohane D. S. (2008). Hydrogels in drug delivery: progress and challenges. Polymer 49, 1993–2007. 10.1016/j.polymer.2008.01.027

[B7] Hong S. M. Sycks D. Chan H. F. Lin S. T. Lopez G. P. Guilak F. (2015). 3D printing of highly stretchable and tough hydrogels into complex, cellularized structures. Adv. Mater. 27, 4035–4040. 10.1002/adma.201501099 26033288 PMC4849481

[B8] Kahn J. S. Ruiz R. C. H. Sureka S. Peng S. M. Derrien T. L. An D.. (2016). DNA microgels as a platform for cell-free protein expression and display. Biomacromolecules 17, 2019–2026. 10.1021/acs.biomac.6b00183 27112709

[B9] Li J. B. Liu H. C. Wang C. Huang G. S. (2017). A facile method to fabricate hybrid hydrogels with mechanical toughness using a novel multifunctional cross-linker. RSC Adv. 7, 35311–35319. 10.1039/C7RA05645A

[B10] Lim H. J. Park Y. J. Jang Y. J. Choi J. E. Oh J. Y. Park J. H.. (2016). Cell-free synthesis of functional phospholipase A1 from *Serratia* sp. Biotechnol. Biofuels 9:159. 10.1186/s13068-016-0563-5 27478501 PMC4966862

[B11] Nishikawa M. Ogawa K. Umeki Y. Mohri K. Kawasaki Y. Watanabe H.. (2014). Injectable, self-gelling, biodegradable, and immunomodulatory DNA hydrogel for antigen delivery. Control. Release 180, 25–32. 10.1016/j.jconrel.2014.02.001 24530618

[B12] Oh K. S. Oh J. S. Choi H. S. Bae Y. C. (1998). Effect of cross-linking density on swelling behavior of NIPA gel particles. Macromolecules 31, 7328–7335. 10.1021/ma971554y

[B13] Pan S. F. Luo S. Li S. Lai Y. S. Geng Y. Y. He B.. (2013). Ultrasound accelerated gelation of novel L-lysine based hydrogelators. Chem. Commun. 49, 8045–8047. 10.1039/c3cc44767g 23903991

[B14] Park N. Kahn J. S. Rice E. J. Hartman M. R. Funabashi H. Xu J. F.. (2009a). High-yield cell-free protein production from P-gel. Nat. Protoc. 4, 1759–1770. 10.1038/nprot.2009.174 20010927

[B15] Park N. Um S. H. Funabashi H. Xu J. F. Luo D. (2009b). A cell-free protein-producing gel. Nat. Mater. 8, 432–437. 10.1038/nmat2419 19329993

[B16] Pugia M. J. Lott J. A. (2005). Pathophysiology and diagnostic value of urinary trypsin inhibitors. Clin. Chem. Lab. Med. 43, 1–16. 10.1515/CCLM.2005.001 15653436

[B17] Rubinstein M. Colby R. H. (2003). Polymer Physics. Vol. 23 New York, NY: Oxford University Press.

[B18] Schoborg J. A. Clark L. G. Choudhury A. Hodgman C. E. Jewett M. C. (2016). Yeast knockout library allows for efficient testing of genomic mutations for cell-free protein synthesis. Synth. Syst. Biotechnol. 1, 2–6. 10.1016/j.synbio.2016.02.004 29062921 PMC5640588

[B19] Stech M. Broedel A. K. Quast R. B. Sachse R. Kubick S. (2013). Cell-free systems: functional modules for synthetic and chemical biology. Adv. Biochem. Eng. Biotechnol. 137, 67–102. 10.1007/10_2013_185 23576054

[B20] Thoring L. Dondapati S. K. Stech M. Wuestenhagen D. A. Kubick S. (2017). High-yield production of “difficult-to-express” proteins in a continuous exchange cell-free system based on CHO cell lysates. Sci. Rep. 7:11710. 10.1038/s41598-017-12188-8 28916746 PMC5601898

[B21] Wei Z. Zhao J. Y. Chen Y. M. Zhang P. B. Zhang Q. Q. (2016). Self-healing polysaccharide-based hydrogels as injectable carriers for neural stem cells. Sci. Rep. 6:12. 10.1038/srep37841 27897217 PMC5126669

[B22] Yang D. Peng S. Hartman M. R. Gupton-Campolongo T. Rice E. J. Chang A. K.. (2013). Enhanced transcription and translation in clay hydrogel and implications for early life evolution. Sci. Rep. 3:3165. 10.1038/srep03165 24196527 PMC3819617

[B23] Yang H. Liu H. Kang H. Tan W. (2008). Engineering target-responsive hydrogels based on aptamer - target interactions. Am. Chem. Soc. 130:6320. 10.1021/ja801339w 18444626 PMC2757630

[B24] Zawada J. F. Yin G. Steiner A. R. Yang J. Naresh A. Roy S. M.. (2011). Microscale to manufacturing scale-up of cell-free cytokine production-a new approach for shortening protein production development timelines. Biotechnol. Bioeng. 108, 1570–1578. 10.1002/bit.23103 21337337 PMC3128707

