# Supplementary material for: A PEGDA/DNA Hybrid Hydrogel for Cell-Free Protein Synthesis
Source: Front Chem. 2020 Feb 18;8:28. doi: 10.3389/fchem.2020.00028 (PMC7039859; doi:10.3389/fchem.2020.00028)
Supplement: Supplementary file 1 [file Data_Sheet_1.pdf]

Supplementary Material

Supplementary Material

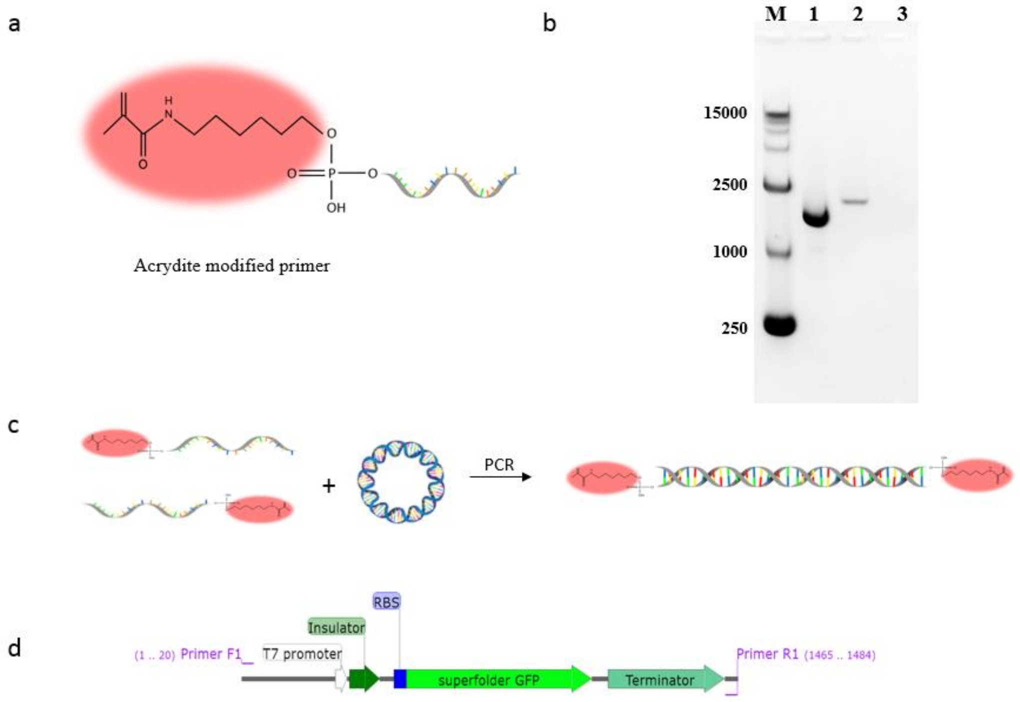

Fig. S1 Preparation of DNA for PEGDA/DNA hybrid hydrogel. (a) Schematic of 5'-acrydite modified primer. (b) Gel electrophoresis image of PCR amplified DNA product. Lane 1 is the DNA product amplified with primers without 5'-acrydite modification. Lane 2 is the DNA product amplified with 5'-acrydite modified primers. All initial experiment parameters and the gel electrophoresis loading volumes were same for lane 1 and 2. However, the band density of DNA product amplified by 5'-acrydite modified primers is lower than that without 5'-acrydite modification. This was possibly due to the fact that the incorporation of acrydite groups on primers lowered the PCR amplification efficiency and thus decreased the DNA product yield of the reaction. (c) Illustration of PCR amplification. The reaction yield a linear DNA with acrydite modification on both ends. (d) Illustration of transcription elements on amplified linear DNA.

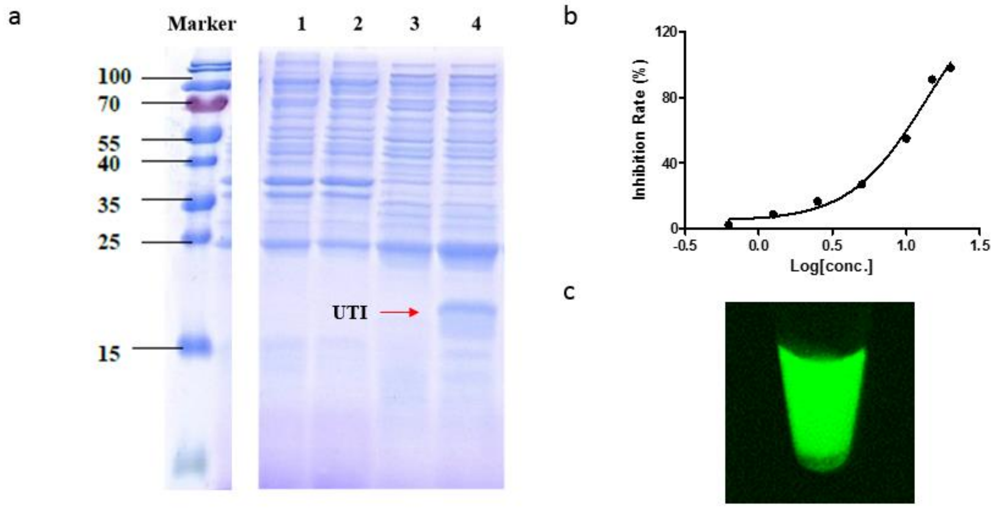

Fig. S2 Functional protein expression by hydrogel phased CFPS. (a) SDS-PAGE image of the UTI expression CFPS samples. Lane 1 & 3, precipitate and supernatant of blank control lysate; lane 2 & 4, precipitate and supernatant of UTI expression lysate. (b) Protease inhibition activity of UTI expressed by hydrogel phased CFPS. (c) Fluorescent image of sfGFP expressed by hydrogel phased CFPS.

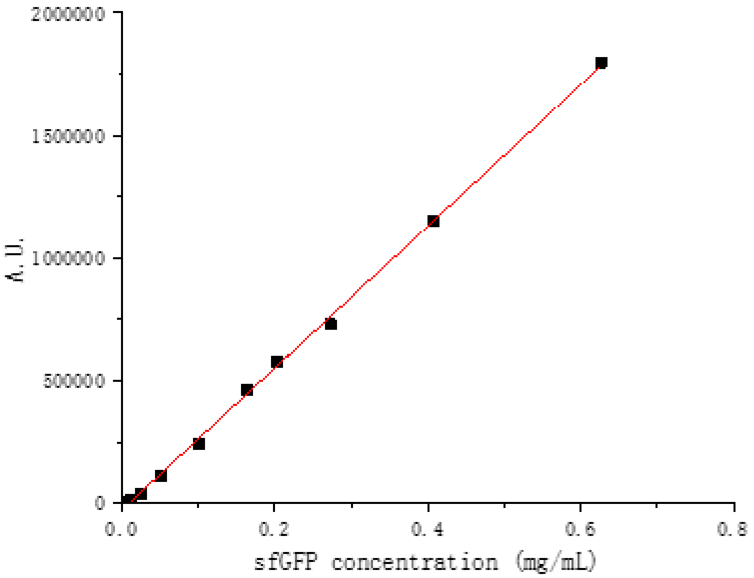

Fig. S3 Fluorescent protein sfGFP standard curve
